# PREDAC-CNN: predicting antigenic clusters of seasonal influenza A viruses with convolutional neural network

**DOI:** 10.1093/bib/bbae033

**Published:** 2024-02-10

**Authors:** Jing Meng, Jingze Liu, Wenkai Song, Honglei Li, Jiangyuan Wang, Le Zhang, Yousong Peng, Aiping Wu, Taijiao Jiang

**Affiliations:** State Key Laboratory of Common Mechanism Research for Major Diseases, Suzhou Institute of Systems Medicine, Chinese Academy of Medical Sciences & Peking Union Medical College, Suzhou 215123, Jiangsu, China; State Key Laboratory of Common Mechanism Research for Major Diseases, Suzhou Institute of Systems Medicine, Chinese Academy of Medical Sciences & Peking Union Medical College, Suzhou 215123, Jiangsu, China; College of Computer Science, Sichuan University, Chengdu 610065, China; Beijing Cloudna Technology Company, Limited, Beijing 100029, China; Guangzhou National Laboratory, Guangzhou 510005, China; College of Computer Science, Sichuan University, Chengdu 610065, China; College of Biology, Hunan University, Changsha 410082, China; State Key Laboratory of Common Mechanism Research for Major Diseases, Suzhou Institute of Systems Medicine, Chinese Academy of Medical Sciences & Peking Union Medical College, Suzhou 215123, Jiangsu, China; State Key Laboratory of Common Mechanism Research for Major Diseases, Suzhou Institute of Systems Medicine, Chinese Academy of Medical Sciences & Peking Union Medical College, Suzhou 215123, Jiangsu, China; Guangzhou National Laboratory, Guangzhou 510005, China; State Key Laboratory of Respiratory Disease, the First Affiliated Hospital of Guangzhou Medical University, Guangzhou Medical University, Guangzhou, 510120, China

**Keywords:** seasonal influenza A viruses, HA sequence, antigenic variant, antigenic cluster, convolutional neural network

## Abstract

Vaccination stands as the most effective and economical strategy for prevention and control of influenza. The primary target of neutralizing antibodies is the surface antigen hemagglutinin (HA). However, ongoing mutations in the HA sequence result in antigenic drift. The success of a vaccine is contingent on its antigenic congruence with circulating strains. Thus, predicting antigenic variants and deducing antigenic clusters of influenza viruses are pivotal for recommendation of vaccine strains. The antigenicity of influenza A viruses is determined by the interplay of amino acids in the HA1 sequence. In this study, we exploit the ability of convolutional neural networks (CNNs) to extract spatial feature representations in the convolutional layers, which can discern interactions between amino acid sites. We introduce PREDAC-CNN, a model designed to track antigenic evolution of seasonal influenza A viruses. Accessible at http://predac-cnn.cloudna.cn, PREDAC-CNN formulates a spatially oriented representation of the HA1 sequence, optimized for the convolutional framework. It effectively probes interactions among amino acid sites in the HA1 sequence. Also, PREDAC-CNN focuses exclusively on physicochemical attributes crucial for the antigenicity of influenza viruses, thereby eliminating unnecessary amino acid embeddings. Together, PREDAC-CNN is adept at capturing interactions of amino acid sites within the HA1 sequence and examining the collective impact of point mutations on antigenic variation. Through 5-fold cross-validation and retrospective testing, PREDAC-CNN has shown superior performance in predicting antigenic variants compared to its counterparts. Additionally, PREDAC-CNN has been instrumental in identifying predominant antigenic clusters for A/H3N2 (1968–2023) and A/H1N1 (1977–2023) viruses, significantly aiding in vaccine strain recommendation.

## INTRODUCTION

Seasonal influenza viruses are responsible for millions of infections and hundreds of thousands of deaths annually, affecting primarily the elderly and children worldwide [1]. Vaccination is recognized as the most effective and cost-efficient strategy for preventing and controlling influenza [2]. To this end, the World Health Organization (WHO) has established National Influenza Centers worldwide, tasked with monitoring antigenic variants and advising on influenza vaccine selections [3]. Despite these concerted global efforts, the challenge of vaccine recommendation remains, as reflected in the varying effectiveness of vaccines [4–6]. The surface antigen hemagglutinin (HA), which is the main target of neutralizing antibodies [7], is prone to mutations that lead to antigenic drift or shift [8]. The efficacy of a vaccine is influenced by the antigenic resemblance between the vaccine strain and the circulating strains [9]. Consequently, accurately predicting antigenic variants and comprehensively understanding the antigenic evolutionary patterns of influenza viruses are indispensable for the effective recommendation of vaccine strains [10].

The experimental identification of antigenic variants predominantly relies on the hemagglutination inhibition (HI) assay, which is known for being both time-consuming and labor-intensive [11]. Smith *et al*. utilized a modified version of metric multidimensional scaling (MDS) to construct an antigenic map for influenza A/H3N2 viruses (1968–2003), revealing that these viruses undergo antigenic evolution in a cluster-wise manner [12]. Subsequent efforts have focused on the development of statistical and machine learning–based models to predict antigenic evolution [13–19]. For instance, we developed the Naïve Bayes classifier PREDAC-H3, designed to predict antigenic variants and deduce antigenic clusters for influenza A/H3N2 viruses [14]. This model considered three types of features: the number of mutations on five epitopes, physicochemical properties of amino acids on surface residues and changes in amino acids within the receptor-binding region and at glycosylation sites. The antigenicity of influenza viruses is influenced by the interactions among amino acids in the HA1 sequence [20]. However, these existing models only take into account a limited number of amino acid sites and are unable to effectively weigh the combined effects of point mutations in the HA1 sequence on antigenicity. As a result, they tend to produce inconsistent predictions [19].

Deep neural networks, with their capacity to extract spatial feature representations and capture interactions at genomic sites, have found successful applications in bioinformatics [21–23]. In the realm of detecting influenza antigenic variants, Lee *et al.* [20] pioneered the use of a convolutional neural network (CNN)–based model for predicting the antigenicity and recommending vaccines for influenza A/H3N2 viruses. This innovation was followed by the development of the FLU [24] and IAV-CNN [25] models. The IAV-CNN model utilized splittings and embeddings to segment the HA1 sequence into shifted, overlapping residue fragments with a window size of 3 and a stride size of 1. However, this approach of splittings and embeddings disrupted the spatial structure of the HA1 sequence, potentially leading to the extraction of misleading local features by the CNN. Moreover, the redundant physicochemical feature representations in these three CNN-based models increased their parameter count, which could lead to a deficiency in the quantity of training samples, affecting the models’ performance.

To overcome the limitations of existing approaches, we introduce PREDAC-CNN for predicting the antigenic evolution of seasonal influenza A viruses. PREDAC-CNN is engineered to create a spatially oriented representation of the HA1 sequence, specifically designed for the convolutional architecture of the CNN model. This design allows for an in-depth exploration of the interactions among amino acids in the HA1 sequence. Distinctively, PREDAC-CNN focuses on physicochemical features that are critical for determining the antigenicity of influenza viruses, rather than relying on redundant amino acid embeddings. Consequently, PREDAC-CNN is adept at capturing the intricate interactions at amino acid sites in the HA1 sequence and analyzing the collective impact of point mutations on antigenic variations. Through 5-fold cross-validations and retrospective testing, PREDAC-CNN has proven to outperform its counterparts in accurately predicting antigenic variants of seasonal influenza A viruses. Additionally, PREDAC-CNN successfully identifies dominant antigenic clusters for influenza A/H3N2 (1968–2023) and A/H1N1 (1977–2023) viruses, significantly advancing the process of vaccine strain recommendation.

## MATERIALS AND METHODS

### Datasets

HI assay data for influenza A/H3N2 and A/H1N1 viruses were gathered from various international sources, including the WHO, the European Centre for Disease Prevention and Control and the US Food and Drug Administration (as detailed in the Data Collection of Supplementary Material available online at http://bib.oxfordjournals.org/). Corresponding HA1 sequences were downloaded from the Influenza Research Database [26], the Global Initiative on Sharing All Influenza Data [27] and the NCBI Influenza Virus Database [28]. We performed sequence deduplication, retaining only strain pairs with a maximum of 15 amino acid substitutions. Consequently, our dataset comprised a total of 8856 strain pairs spanning 1968 to 2022 for influenza A/H3N2 viruses and 876 strain pairs from 1951 to 2022 for influenza A/H1N1 viruses. This dataset forms the foundation for the complete antigenic relationship analysis used in the development of PREDAC-CNN.

For the purpose of inferring antigenic clusters, we downloaded HA1 sequences of influenza A/H3N2 (1968–2023) and A/H1N1 (1977–2023) viruses from the Global Initiative on Sharing All Influenza Data [27]. To reduce sequence redundancy, we employed CD-HIT [29], which resulted in 2591 representative HA1 sequences for influenza A/H3N2 (with a similarity threshold of 0.985) and 2598 representative HA1 sequences for influenza A/H1N1 (with a similarity threshold of 0.988) viruses. Additionally, we investigated the impact of sequence redundancy reduction on the inference of antigenic clusters by conducting six rounds of random sampling without replacement from each CD-HIT cluster. This process yielded varying counts of HA1 sequences for influenza A/H3N2 (2594, 2595, 2597, 2596, 2589 and 2594) and A/H1N1 (2596, 2598, 2597, 2597, 2597 and 2597) viruses. We labeled these seven sets of HA1 sequences for each virus type as sample-1 through sample-7. Phylogenetic trees for these sequences were then constructed using FastTree [30].

### Characterization of antigenic relationships of strain pairs

Antigenic distance ${\mathrm{D}}_{ij}$ between paired strains is represented by the Archetti–Horsfall distance [31] as follows:


(1) \begin{equation*} {\mathrm{D}}_{ij}=\sqrt{\frac{{\mathrm{H}}_{ii}\times{\mathrm{H}}_{jj}}{{\mathrm{H}}_{ij}\times{\mathrm{H}}_{ji}}} \end{equation*}

where ${\mathrm{H}}_{ij}$denotes the HI titer of strain i required to inhibit cell agglutination caused by strain $j$. Two strains are classified as antigenic variants if their antigenic distance, ${\mathrm{D}}_{ij}$, is equal to or exceeds 4. If the distance is less, they are considered antigenically similar [32]. Consequently, among the 8856 strain pairs analyzed for influenza A/H3N2 viruses, 4925 pairs were categorized as antigenically distinct (positive) and 3931 as antigenically similar (negative). For the 876 strain pairs of influenza A/H1N1 viruses, 362 pairs were defined as antigenically distinct (positive), and 514 pairs as antigenically similar (negative). Tables 1 and 2 detail the distributions of these antigenically distinct and similar strain pairs for influenza A/H3N2 and A/H1N1 viruses, respectively. In these tables, for any given year, a strain pair is composed of one strain isolated in that specific year and the other strain isolated in or before that year.

**Table 1:** The distributions of antigenically distinct strain pairs and antigenically similar strain pairs for each year for influenza A/H3N2 viruses

Year	Antigenically similar	Antigenically distinct	Year	Antigenically similar	Antigenically distinct
1968	3	0	1996	203	307
1969	7	2	1997	129	184
1970	11	0	1998	34	41
1971	35	4	1999	66	21
1972	12	40	2000	13	4
1973	23	46	2001	75	4
1974	45	26	2002	44	12
1975	22	28	2003	64	29
1976	8	44	2004	105	25
1977	24	25	2005	115	61
1979	0	14	2006	80	17
1980	3	5	2007	165	20
1981	6	0	2008	44	11
1982	18	27	2009	42	136
1983	6	0	2010	62	80
1984	6	19	2011	56	11
1985	59	10	2012	47	0
1986	17	0	2013	79	37
1987	16	81	2014	59	39
1988	34	28	2015	14	17
1989	136	132	2016	82	50
1990	55	60	2017	42	61
1991	76	151	2018	12	42
1992	370	567	2019	16	47
1993	617	1458	2020	52	43
1994 1995	254 268	322 471	2021 2022	44 56	35 31

*Note.* For each specific year, a strain pair contains a strain isolated on that year and the other strain isolated on or before that year.

**Table 2:** The distributions of antigenically distinct strain pairs and antigenically similar strain pairs for each year for influenza A/H1N1 viruses

Year	Antigenically similar	Antigenically distinct	Year	Antigenically similar	Antigenically distinct
1951	0	1	2004	19	6
1954	0	2	2005	14	14
1957	0	1	2006	47	82
1978	9	17	2007	29	33
1979	1	5	2008	46	28
1980	2	9	2009	54	20
1982	4	5	2010	29	2
1983	7	14	2011	25	0
1986	2	4	2012	11	11
1988	6	2	2013	27	6
1991	7	1	2014	9	2
1995	5	16	2015	27	3
1996	6	7	2016	20	3
1997	3	1	2017	27	0
1998	2	5	2018	13	0
1999	3	5	2019	15	15
2000	5	7	2020	2	6
2002	21	1	2021	9	20
2003	5	4	2022	3	4

*Note.* For each specific year, a strain pair contains a strain isolated on that year and the other strain isolated on or before that year.

### Development of PREDAC-CNN

**Figure 1:**
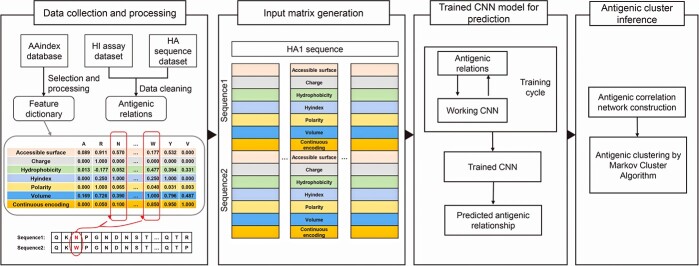
Overview of PREDAC-CNN. In the first step, PREDAC-CNN takes paired HA1 sequences from influenza A/H3N2 or A/H1N1 viruses as input. It generates an input matrix that corresponds to the paired HA1 sequences (sequence 1 and sequence 2) by encoding selected physicochemical features of amino acids using the feature dictionary. Subsequently, PREDAC-CNN utilizes the CNN model to assess the input matrix and infer the antigenic relationship between the paired strains of influenza A/H3N2 or A/H1N1 viruses. In the second step, PREDAC-CNN connects strains exhibiting similar antigenicity to construct an antigenic correlation network and performs antigenic clustering using the MCL method.

The workflow of PREDAC-CNN is illustrated in Figure 1. The inputs are paired HA1 sequences of influenza A/H3N2 or A/H1N1 viruses. For the purpose of training the CNN model, antigenic relationships are established by integrating these paired HA1 sequences with their corresponding antigenic relationships. PREDAC-CNN then encodes the selected physicochemical properties of amino acids, based on our predefined feature dictionary. Subsequently, an input matrix for the paired HA1 sequences (sequence 1 and sequence 2) is generated. This matrix is specifically tailored as a spatially oriented representation suitable for convolutional operations. The encoded physicochemical features of sequence 2 are integrated with those of sequence 1. Utilizing this input matrix, PREDAC-CNN employs the CNN model to predict the antigenic relationship between the paired influenza A/H3N2 or A/H1N1 virus strains. In the next phase, PREDAC-CNN constructs an antigenic correlation network based on these predicted relationships and deduces antigenic clusters. The following section delves into the detailed functioning of PREDAC-CNN.

### Input matrix generation

The antigenicity of influenza viruses is intricately linked to the interactions among amino acids in the HA1 sequence. In our approach, we selected six physicochemical properties of amino acids that are pivotal in determining antigenicity. These properties are accessible surface, charge, hydrophobicity, hyindex (which indicates the potential of an amino acid to act as a hydrogen-bond donor and/or acceptor), polarity and volume [13, 14]. The AAindex1 database houses multiple entries for each of these six physicochemical properties (as detailed in the Data Collection of Supplementary Material available online at http://bib.oxfordjournals.org/) [13, 33]. To enhance model performance, it is essential to scale these values to fall within the range of [−1, 1]. To achieve this, we employed data normalization, processing the values for each of the six properties using the following formula [34]:


(2) \begin{equation*} {X}_{\mathrm{norm}}=\left\{\begin{array}{cc}\frac{X_i}{\max \left(\left|{X}_{\mathrm{min}}\right|,\left|{X}_{\mathrm{max}}\right|\right)}& \mathrm{if}\left({X}_{\mathrm{min}}<0\right)\\{}\frac{X_i-{X}_{\mathrm{min}}}{X_{\mathrm{max}}-{X}_{\mathrm{min}}}& \mathrm{if}\left({X}_{\mathrm{min}}>0\right)\end{array}\right\} \end{equation*}

To further determine the most effective entry for each physicochemical property, we adopted a meticulous approach. For influenza A/H3N2 and A/H1N1 viruses, we created a matrix of size 329 × 4 and 327 × 4, respectively, for each entry of every physicochemical property. The selection of the optimal entry was based on the highest F_1_ score achieved during validations for each specific property. Take, for instance, the physicochemical property of polarity, which has three entries in the AAindex1 database. To identify the best-suited entry for polarity in the context of influenza A/H3N2 viruses, we generated three distinct input matrices, each corresponding to one of the three entries. In these matrices, designed for the paired HA1 sequences (sequence 1 and sequence 2), the 329 columns represent the amino acid sites in the HA1 sequence. Meanwhile, the four rows in each column correspond to the specific amino acid and the entry of its polarity property for the paired sequences. The entry for polarity that achieved the highest F_1_ score in the validations was selected as the optimal entry. Our feature dictionary, as a result, comprises six physicochemical features with their respective optimal entries, along with a continuous encoding scheme for the 20 amino acids, tailored for influenza A/H3N2 and A/H1N1 viruses (as detailed in Supplementary Tables S1 and S2 available online at http://bib.oxfordjournals.org/).

Subsequently, PREDAC-CNN integrates and encodes the six selected physicochemical features into the input matrix for the paired HA1 sequences, preparing it for processing by the CNN model. This step is crucial for thoroughly investigating the interactions between amino acid sites in the HA1 sequence. The construction of the input matrix, based on the feature dictionary, results in a spatially oriented representation of the HA1 sequence. This matrix comprises 14 rows, with 7 rows allocated for each sequence, and either 329 columns for influenza A/H3N2 viruses or 327 columns for influenza A/H1N1 viruses. Within each column, the first seven rows represent the amino acid at that specific site and its six physicochemical features for sequence 1, while the last seven rows do the same for sequence 2. This method of representing amino acid features along with their physicochemical properties in the matrix is referred to as the ‘7-features’ encoding.

### The CNN model for prediction

The input matrix, representing paired HA1 sequences from two different strains, is inherently two-dimensional. This complexity presents significant challenges for traditional machine learning algorithms, especially when dealing with such high-dimensionality data. However, CNN models have demonstrated remarkable success in various fields where inputs are characteristically high-dimensional [21–23]. Leveraging these proven capabilities, we utilize the convolutional computation within CNN models to learn abstract feature representations in higher-level feature layers.

We utilize the CNN model to ascertain the antigenic relationship between two strains of either influenza A/H3N2 or A/H1N1 viruses. The detailed architecture of this CNN model is presented in Supplementary Figure S1 available online at http://bib.oxfordjournals.org/. For influenza A/H3N2 viruses, the input matrix size is 14 × 329, while for influenza A/H1N1 viruses, it is 14 × 327. The model conducts one-dimensional convolutional computations, as the mappings of the physicochemical features of amino acids in a HA1 sequence pair lack spatial representation. The convolutional computations use a kernel/filter size of 3 and a stride of 1, which facilitates the extraction of features from both downstream and upstream amino acid sites surrounding a specific site in the HA1 sequence. After every two consecutive convolutional computations, maxpooling is applied to distill the most significant mappings of physicochemical features within small spatial amino acid regions. The maxpooling employs a kernel/filter size of 2 and a stride of 2. Ultimately, based on the abstract feature representations derived from the preceding layers, the classification layer carries out the final step of classification to determine the antigenic relationship of a strain pair of influenza A/H3N2 or A/H1N1 viruses.

### Antigenic cluster inference

Our approach to inferring antigenic clusters builds on methodologies established in our previous research [14, 16]. Once the CNN model predicts the antigenic relationship between paired strains, PREDAC-CNN forms connections between strains exhibiting similar antigenicity, thereby constructing an antigenic correlation network. Subsequent to this network formation, antigenic clustering is conducted using the Markov Cluster Algorithm (MCL) method. A critical factor in this process is the threshold main inflation value used in the MCL method, which significantly influences the granularity of the clusters [14]. For the purpose of inferring antigenic clusters, we select the threshold main inflation value corresponding to the onset of the first plateau observed in the cluster size curve, as depicted in Supplementary Figure S2 available online at http://bib.oxfordjournals.org/.

### Experimental design

In order to assess the effectiveness of PREDAC-CNN in predicting antigenic relationships, we conducted two distinct types of benchmarking experiments. The first type involved evaluating how different feature encodings impact the performance of the CNN model and determining if the CNN model surpasses traditional machine learning algorithms. To this end, we incorporated three other state-of-the-art feature encodings: AAindex-PCA [24], ESM-2 [36] and iFeatureOmega [37]. Additionally, we tested four machine learning algorithms, namely, logistic regression (LR), K-nearest neighbor (KNN), neural network (NN) and support vector machine (SVM). The methodology for designing these three additional feature encodings is detailed in the ‘Feature Encodings’ section of the Supplementary Material available online at http://bib.oxfordjournals.org/. For each feature encoding, we conducted a comparative analysis between PREDAC-CNN and the four aforementioned machine learning algorithms. In the second type of benchmarking experiment, we extended our comparison to include PREDAC-CNN and five state-of-the-art competitors in the field. These competitors are IAV-CNN [25], PREDAV-FluA [17], Lee [38], lees [39] and PREDAC-H3 [14] for influenza A/H3N2 viruses, along with PREDAC-H1 [40] for influenza A/H1N1 viruses.

In each benchmarking experiment type, we initially did 5-fold cross-validations. The comprehensive dataset of antigenic relationships was divided into distinct segments: training sets for model fitting, validation sets for the optimization of model hyperparameters and testing sets for the purpose of evaluative experiments. Subsequently, we conducted retrospective testing, particularly in the context of vaccine recommendation applications. The effectiveness of a vaccine is dependent on the antigenic similarity between the vaccine strain and the circulating strains of a particular season [35]. A crucial aspect of vaccine recommendation is to determine the antigenic relationship between the vaccine strain and the strains circulating in the upcoming influenza season. Therefore, to align with the practical scenario of vaccine recommendation, retrospective testing was implemented to evaluate the models’ predictive performances.

In these retrospective tests, the isolation dates of each strain pair were considered. Due to the scarcity of strain pairs prior to 2005, the tests focused on strain pairs isolated between 2006 and 2022 for both influenza A/H3N2 and A/H1N1 viruses. For the A/H3N2 viruses, the models were trained using antigenic data from 1968 to the year preceding a specified target year and then tested on data from the target year. Similarly, for the A/H1N1 viruses, the models were trained on data from 1951 to the year preceding the target year and subsequently tested on the target year’s data. For instance, in the case of A/H3N2 viruses with 2007 as the target year for testing, the models were trained on antigenic relations spanning from 1968 to 2006. Each strain pair for the test year 2007 included one strain isolated in 2007 and the other strain isolated on or before 2007.

To assess and compare the performance of the models in predicting antigenic relationships, we employed two key statistical measures: precision and recall. These metrics are defined in the following manner:


(3) \begin{equation*} \mathrm{Precision}=\frac{\mathrm{TP}}{\mathrm{TP}+\mathrm{FP}} \end{equation*}

(4) \begin{equation*} \mathrm{Recall}=\frac{\mathrm{TP}}{\mathrm{TP}+\mathrm{FN}} \end{equation*}

where TP (true positive) denotes the number of strain pairs correctly predicted as antigenically distinct. FP (false positive) refers to the strain pairs incorrectly predicted as antigenically distinct. TN (true negative) represents the strain pairs correctly identified as antigenically similar. FN (false negative) is the count of strain pairs incorrectly classified as antigenically similar. The *F*_1_ score, serving as a measure of a model’s overall performance, is the harmonic mean of precision and recall. Its formula is defined as follows:


(5) \begin{equation*} {\mathrm{F}}_1=\frac{2\times \mathrm{precision}\times \mathrm{recall}}{\mathrm{precision}+\mathrm{recall}} \end{equation*}

## RESULTS

### Overview of PREDAC-CNN

In this study, we exploit the CNN’s ability to discern interactions between amino acid sites and develop PREDAC-CNN to predict antigenic evolution of seasonal influenza A viruses. The inputs are paired HA1 sequences of influenza A/H3N2 or A/H1N1 viruses. PREDAC-CNN then integrates and encodes the six selected physicochemical features into the input matrix for the paired HA1 sequences. This method of representing amino acid features along with their physicochemical properties in the input matrix is referred to as the ‘7-features’ encoding. Utilizing this input matrix, PREDAC-CNN employs the CNN model to predict the antigenic relationship between the paired influenza A/H3N2 or A/H1N1 virus strains. In the next phase, PREDAC-CNN forms connections between strains exhibiting similar antigenicity, thereby constructing an antigenic correlation network. Subsequent to this network formation, antigenic clustering is conducted using the MCL method.

### Performance evaluation of PREDAC-CNN

In order to thoroughly compare the performance of various models in predicting antigenic relationships, we implemented two distinct types of benchmarking experiments. The first type of benchmarking experiment involved evaluating how different feature encodings impact the performance of the CNN model and determining if the CNN model surpasses traditional machine learning algorithms. In this process, we incorporated three other state-of-the-art feature encodings and tested four machine learning algorithms. For each feature encoding, we conducted a comparative analysis between PREDAC-CNN and the four machine learning algorithms.

**Figure 2:**
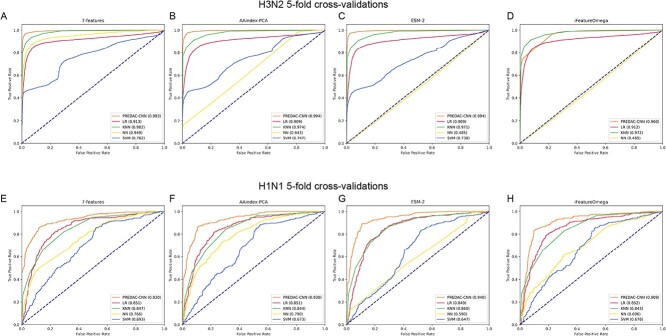
ROC curves for 5-fold cross-validations of PREDAC-CNN and four machine learning models with four feature encodings. ROC curves for feature encoding of 7-features (**A**), AAindex-PCA (**B**), ESM-2 (**C**) and iFeatureOmega (**D**) on influenza A/H3N2 viruses. ROC curves for feature encoding of 7-features (**E**), AAindex-PCA (**F**), ESM-2 (**G**) and iFeatureOmega (**H**) on influenza A/H1N1 viruses.

**Figure 3:**
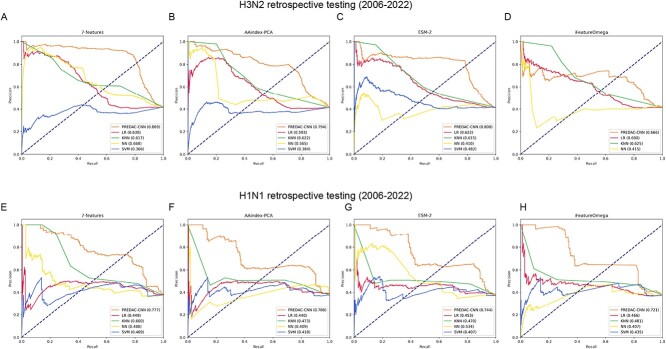
PRROC curves for retrospective testing of PREDAC-CNN and four machine learning models with four feature encodings. PRROC curves for feature encoding of 7-features (**A**), AAindex-PCA (**B**), ESM-2 (**C**) and iFeatureOmega (**D**) on influenza A/H3N2 viruses. PRROC curves for feature encoding of 7-features (**E**), AAindex-PCA (**F**), ESM-2 (**G**) and iFeatureOmega (**H**) on influenza A/H1N1 viruses.

Among the four feature encodings evaluated, PREDAC-CNN and the four machine learning models exhibited their optimal performances with the 7-features encoding, as depicted in Figure 2A–H. Similar to the outcomes observed in the 5-fold cross-validation experiments, PREDAC-CNN and the four machine learning models achieved their best performances using the 7-features encoding among the four feature encodings for retrospective testing, as shown in Figure 3A–H. These outcomes underscore the superiority of using antigenicity-determining physicochemical features over general-purpose physicochemical feature encodings.

Notably, PREDAC-CNN demonstrated superior performances compared to the four machine learning models when using the best feature encoding (as shown in Figures 2A and E and 3A and E). This highlights the effectiveness of a spatially oriented representation of the HA1 sequence, tailored to the convolutional architecture of the CNN model. Such an approach efficiently facilitates the exploration of amino acid interactions and scales appropriately to assess the collective impact of point mutations in the HA1 sequence on antigenicity.

### Comparison of PREDAC-CNN with state-of-the-art models

**Figure 4:**
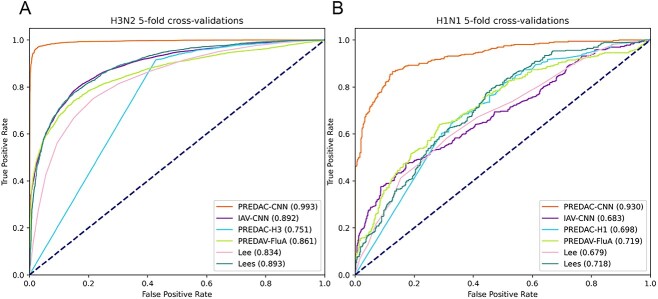
ROC curves for 5-fold cross-validations of PREDAC-CNN and its five competitors. ROC curves on influenza A/H3N2 viruses (**A**) and influenza A/H1N1 viruses (**B**).

Then, we conducted the second type of benchmarking experiment, where we extended our comparison to compare PREDAC-CNN with five state-of-the-art competitors in the field. For 5-fold cross-validations, PREDAC-CNN exhibited the best performance in the comparison against five competitive models (as shown in Figure 4A and B, with detailed data in Supplementary Tables S3–S12 available online at http://bib.oxfordjournals.org/). This superior performance of PREDAC-CNN, along with that of the five competitors, was particularly pronounced for influenza A/H3N2 viruses (illustrated in Figure 4A, with detailed data in Supplementary Tables S3–S7 available online at http://bib.oxfordjournals.org/). This can be attributed to the substantial training dataset available for this virus type, encompassing 8856 strain pairs. In contrast, the dataset for influenza A/H1N1 viruses consisted of only 876 strain pairs, posing a significant challenge for all the models. Consequently, the comparatively lower performance of all models on influenza A/H1N1 viruses, relative to A/H3N2 viruses, is understandable given the markedly smaller size of the available antigenic relation data for A/H1N1.

For retrospective testing, it encompassed a total of 17 subsets for both influenza A/H3N2 and A/H1N1 viruses, corresponding to different testing years. During the retrospective testing, we encountered instances where the number of antigenically distinct strain pairs was zero. Specifically, this occurred in the 2012 testing subset for influenza A/H3N2 viruses (as shown in Table 1) and in the 2011, 2017 and 2018 testing subsets for influenza A/H1N1 viruses (Table 2). Due to the absence of antigenically distinct pairs in these subsets, it was not possible to calculate the AUPRC (area under the precision–recall ROC curve) values for PREDAC-CNN and its five competitor models.

**Figure 5:**
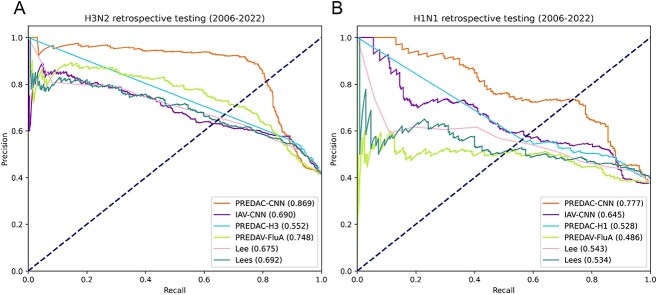
PRROC curves for retrospective testing of PREDAC-CNN and its five competitors. PRROC curves on influenza A/H3N2 viruses (**A**) and influenza A/H1N1 viruses (**B**).

As was the case with 5-fold cross-validations, PREDAC-CNN demonstrated superior performance over its five competitor models for retrospective testing, as depicted in Figure 5A and B. For the remaining 16 subsets of influenza A/H3N2 viruses and 14 subsets of influenza A/H1N1 viruses, PREDAC-CNN achieved the highest AUPRC values in 10 of these testing subsets, as detailed in Supplementary Tables S13 and S14 available online at http://bib.oxfordjournals.org/. Notably, the number of training samples increased with each successive testing year, as indicated in Tables 1 and 2. This increase in training data allowed the models to more effectively learn the correlations between point mutations and antigenic variants, thereby enhancing their ability to filter out inaccurate predictions during the inference process. Consequently, it was anticipated that the performance of PREDAC-CNN and its competitors would improve with each testing year, reflecting the models’ increasing proficiency in accurately predicting antigenic relationships.

To illustrate the impact of antigenic drift on vaccine effectiveness, we utilized PREDAC-CNN to analyze the antigenic relationships between circulating strains and the WHO-recommended vaccine strains across three influenza seasons in the United States. The findings, presented in Supplementary Figure S3 available online at http://bib.oxfordjournals.org/, indicate that for both influenza A/H3N2 and A/H1N1 viruses, there was a noticeable increase in the percentages of predicted antigenic variants from the 2017–18 season to the 2019–20 season. This trend aligns with the observed decline in vaccine effectiveness during these periods, as reported in references [4–6]. For instance, the effectiveness of the vaccine against influenza A/H1N1 viruses decreased from 62% in the 2017–18 season to just 30% in the 2019–20 season. Corresponding to this drop in vaccine effectiveness, our analysis showed an increase in the percentage of predicted antigenic variants from 1.6% to 18.8%.

### Application of PREDAC-CNN to capture antigenic evolution of influenza A viruses

**Figure 6:**
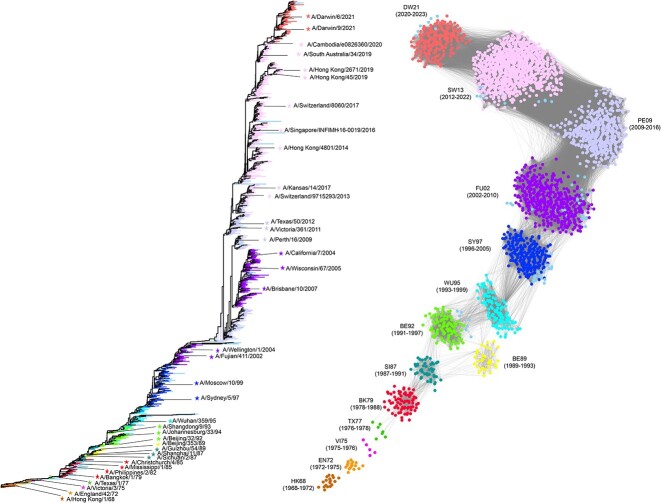
Genetic and antigenic evolution of influenza A/H3N2 viruses. The left panel illustrates genetic evolution, with WHO-recommended vaccine strains denoted by five-pointed stars. The right panel shows antigenic evolution, with antigenic clusters named after abbreviations of the WHO-recommended earliest vaccine strains included in the clusters.

**Figure 7:**
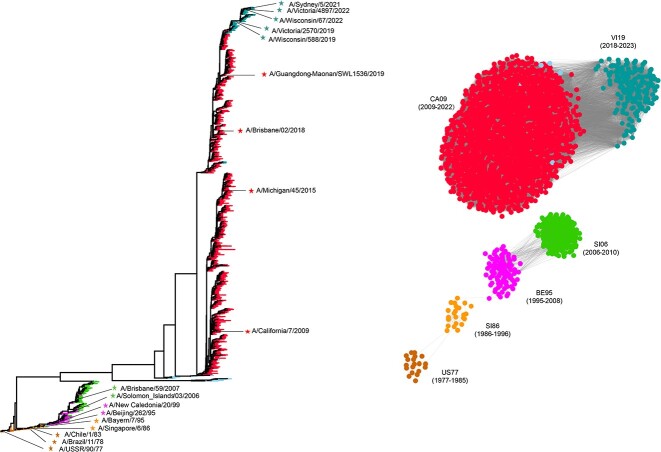
Genetic and antigenic evolution of influenza A/H1N1 viruses. The left panel illustrates genetic evolution, with WHO-recommended vaccine strains denoted by five-pointed stars. The right panel shows antigenic evolution, with antigenic clusters named after abbreviations of the WHO-recommended earliest vaccine strains included in the clusters.

In an effort to comprehensively understand the antigenic evolution of influenza A viruses, we applied PREDAC-CNN to the task of inferring antigenic clusters for both influenza A/H3N2 (spanning 1968–2023) and A/H1N1 (spanning 1977–2023) viruses. Our analysis led to the identification of 14 dominant antigenic clusters for A/H3N2 viruses and 6 antigenic clusters for A/H1N1 viruses, as depicted in Figures 6 and 7. While the genetic evolution of influenza A viruses is a continuous process, our findings further verify that their antigenic evolution tends to follow a pattern of distinct, cluster-wise changes [12]. These antigenic clusters are characterized by serial replacement, where each cluster is succeeded by another comprising antigenically distinct strains. Significantly, the identified antigenic clusters for both A/H3N2 and A/H1N1 viruses displayed consistency across the seven samples in terms of the circulation time of each cluster. This consistency, detailed in Supplementary Tables S15 and S16 available online at http://bib.oxfordjournals.org/, indicates that the reduction of sequence redundancy did not affect the inference of antigenic clusters.

For influenza A/H3N2 viruses, the analysis revealed that the dominant antigenic clusters, which accounted for over 98% of the strains, persisted for durations ranging from 1 to 10 seasons, with an average dominance period of 3.9 years. Notably, there was one cluster that predominated for a single season and two clusters that maintained dominance for as long as 10 seasons. Our analysis accurately identified eleven dominant antigenic clusters for the period from 1968 to 2003, aligning with the findings of Smith *et al*. [12]. Interestingly, the vaccine strain A/California/7/2004 was found to be antigenically similar to both A/Brisbane/10/2007 and A/Fujian/411/2002. PREDAC-CNN effectively grouped these strains into the same cluster, termed FU02. However, this grouping contrasts with the classification in the study by Bedford *et al*. [41], where A/California/7/2004 and A/Brisbane/10/2007 were placed in different clusters. The cluster SW13 was found to include two antigenically distinct pairs of vaccine strains: A/Singapore/INFIMH-16-0019/2016 and A/Hong Kong/45/2019 and A/Switzerland/8060/2017 and A/South Australia/34/2019. This classification was justifiable given that the antigenic distances of these pairs were very close to the threshold value of 4. Additionally, since the antigenic distance between A/Singapore/INFIMH-16-0019/2016 and A/Cambodia/e0826360/2020 was significantly higher at 11.3, and considering that A/Cambodia/e0826360/2020 was antigenically similar to A/Darwin/9/2021, it is plausible that A/Cambodia/e0826360/2020 belonged to the cluster DW21 rather than SW13.

For influenza A/H1N1 viruses, our analysis found that antigenic clusters generally remained dominant for an average of 7.5 years. This suggests a slower evolutionary pace for A/H1N1 viruses compared to A/H3N2 viruses. In our study, all vaccine strains with similar antigenicity were consistently grouped into the same antigenic clusters. Conversely, vaccine strains with distinct antigenicity were typically placed in different clusters. An exception to this was observed with A/Beijing/262/95 and A/New Caledonia/20/99, which were the WHO-recommended vaccine strains grouped in the antigenic cluster BE95 [41, 42]. PREDAC-CNN’s decision to classify these two strains into the same cluster was justified, given that their antigenic distance, based on HI assays, was exactly 4. Compared to influenza A/H3N2 viruses, a higher number of minor antigenic clusters were identified for A/H1N1. These minor clusters included a total of 17 strains and did not encompass any WHO-recommended vaccine strains. This observation aligns with the relatively lower performance of PREDAC-CNN in accurately predicting antigenic variants of A/H1N1 viruses. Notably, the seasonal influenza A/H1N1 viruses circulating from 1977 to 2008 were antigenically distinct from the A/H1N1 pandemic viruses that emerged in 2009. The pandemic viruses exhibited a slower rate of evolution compared to the earlier seasonal strains. Since 2018, a new antigenic cluster, VI19, has emerged, supplanting the preceding antigenic cluster CA09.

To demonstrate the utility of inferring antigenic clusters in guiding vaccine composition changes, we analyzed the dynamic shifts in predicted antigenic clusters of influenza A/H1N1 viruses over three influenza seasons from 2020 to 2022 in the Southern Hemisphere. Our approach involved making vaccine recommendations toward the end of September for each season, as illustrated in Supplementary Figure S4 available online at http://bib.oxfordjournals.org/. The guiding principle for these recommendations was based on the emergence and prevalence of new antigenic clusters. Specifically, in September, if a new antigenic cluster emerged and its prevalence exceeded that of the existing dominant cluster, it was predicted to become dominant in the upcoming season, warranting an update in the vaccine strain. Conversely, if the new cluster did not surpass the old cluster in prevalence, the existing cluster was expected to continue its dominance, negating the need for a vaccine strain update. Applying this criterion led to the recommendation of updating the vaccine strain for the 2022 season, which was then continued for the 2023 season. Notably, these predicted vaccine strains aligned with the WHO’s vaccine strain recommendations.

## DISCUSSION

Accurately predicting antigenic variants and characterizing the antigenic evolution of influenza viruses are fundamental aspects of vaccine strain recommendation. Previous studies employing machine learning and statistical methods mainly focused on specific amino acid sites, such as antigenic epitopes, which have been identified in the evolution of influenza A viruses. However, these approaches lacked the scalability to thoroughly explore the contribution of amino acid interactions within the HA1 sequence to antigenicity [13–19]. More recent studies have turned to deep learning, considering a wide array of physicochemical features. While encompassing a broad spectrum of protein properties, including structure, function and others derived from experimental and high-quality protein structures, it is important to note that these general-purpose features are often resource-intensive and time-consuming to obtain and that they have significantly increased the demands for computational resources and training samples [20, 24, 25]. Moreover, they may introduce redundancy and irrelevance in the context of influenza virus antigenicity. This shift in methodology is evident in the higher computational costs associated with models like IAV-CNN, as detailed in Supplementary Tables S3–S12 available online at http://bib.oxfordjournals.org/. In our study, we chose a targeted approach, focusing exclusively on physicochemical features that are critical in determining antigenicity. We designed a spatially oriented representation of the HA1 sequence that is specifically tailored for the convolutional architecture of the CNN model. This strategic design choice has paid off, as evidenced by the superior performance of PREDAC-CNN in both 5-fold cross-validation trials and retrospective tests.

Overall, the performance metrics of the models in the 5-fold cross-validations were notably better and more consistent compared to those in the retrospective testing (illustrated in Figures 4A and B and 5A and B, with detailed data in Supplementary Tables S3–S14 available online at http://bib.oxfordjournals.org/). For instance, with influenza A/H3N2 viruses, PREDAC-CNN achieved an impressive AUROC (area under the receiver operating characteristic curve) value of 0.993 in the 5-fold cross-validations (Figure 4A). However, during retrospective testing, the AUPRC values for PREDAC-CNN ranged from 0.463 to 1 (Supplementary Table S13 available online at http://bib.oxfordjournals.org/). This variation in performance can be attributed to the nature of influenza A virus evolution. The HA sequence of these viruses undergoes gradual mutations over time. In the context of retrospective testing, the mutation patterns present in the HA1 sequences of the testing subsets were not previously characterized by the models, which were trained on experimentally validated antigenic relations. This lack of prior exposure to specific mutation patterns in the training phase likely affected the models’ accuracy in the retrospective tests. Given the evolutionary dynamics of influenza A viruses and the design of the testing methodologies, the observed discrepancies in model performance between the 5-fold validations and retrospective testing are reasonable and align with the expectations for predictive modeling in evolving viral landscapes.

The substitution rate of the HA1 sequence in influenza A/H3N2 viruses was quantified at 5.7 nucleotides per year [43], with an accompanying average of 3.6 amino acid substitutions per year [12]. Conversely, influenza A/H1N1 viruses exhibited a comparatively slower pace of nucleotide and amino acid substitutions [41, 44]. Antigenic epitopes within the HA1 sequence are subjected to sustained immune-driven selection pressure, resulting in frequent mutations [12, 45]. By means of antigenic cluster inference, we have delineated intricate antigenic evolutionary patterns, thereby enhancing our comprehension of antigenic evolution dynamics. Our findings reveal that influenza A/H1N1 viruses undergo less frequent antigenic cluster replacements in contrast to their A/H3N2 counterparts. This observation further corroborates the notion that influenza A/H1N1 viruses undergo a less rapid antigenic evolution trajectory, complementing prior genetic evolution studies. Furthermore, we have expanded the utility of PREDAC-CNN to illustrate the impact of antigenic drift on vaccine efficacy, as well as to elucidate how the inference of antigenic clusters can aid in the anticipation of shifts in vaccine composition. It should be noted, however, that multiple variables, such as immune imprinting and pre-existing immunity within the population, exert influence on vaccine efficacy [45]. Thus, the proportion of antigenic variants serves as an initial evaluation metric for vaccine effectiveness. In summary, the development of PREDAC-CNN significantly contributes to the realm of influenza surveillance and informs vaccine recommendation strategies.

Due to the notably limited size of the presently accessible antigenic relationship dataset, PREDAC-CNN has yielded suboptimal performance outcomes when applied to influenza A/H1N1 viruses. However, as additional HI assay values for influenza A/H1N1 viruses become accessible in the future, we are committed to enhancing PREDAC-CNN by updating its training dataset. Furthermore, we recognize that transfer learning represents an effective methodology for mitigating the reliance on extensive training data [21, 46]. To this end, we plan to leverage biologically relevant pre-trained models to enhance the performance of PREDAC-CNN.

**Key Points**

- We leverage the advantages of CNNs in extracting spatial feature representations through receptive fields at convolutional layers and in capturing dependencies among amino acid sites. This forms the foundation for our proposal, PREDAC-CNN, which aims to model the antigenic evolution of seasonal influenza A viruses.
- PREDAC-CNN constructs a spatially oriented representation of the HA1 sequence tailored to the convolutional architecture of the CNN model, enabling the exploration of interactions among amino acids within the HA1 sequence.
- PREDAC-CNN considers only physicochemical features that determine antigenicity, avoiding the inclusion of redundant amino acid embeddings.
- PREDAC-CNN effectively captures dependencies among amino acid sites in the HA1 sequence and examines the combined contributions of point mutations in the HA1 sequence to antigenic variations.
- PREDAC-CNN is capable of identifying dominant antigenic clusters among seasonal influenza A viruses.

## Supplementary Material

SupplementaryMaterial_final_bbae033

## Data Availability

Source code of PREDAC-CNN is publicly available at https://github.com/jingmeng-bioinformatics/PREDAC-CNN.

## Author Biographies

**Jing Meng** is an assistant professor at Suzhou Institute of Systems Medicine, Center for Systems Medicine, Chinese Academy of Medical Sciences & Peking Union Medical College, Suzhou, Jiangsu, China. Her research interests are deep learning and bioinformatics.

**Jingze Liu** is doing her PhD degree at Chinese Academy of Medical Sciences & Peking Union Medical College.

**Wenkai Song** is doing his master’s degree at Sichuan University.

**Honglei Li** is a software engineer at Beijing Cloudna Technology Company.

**Jiangyuan Wang** is a postdoctoral researcher at Guangzhou National Laboratory. His research interests are deep learning and bioinformatics.

**Le Zhang** is a professor at Sichuan University. His research interests are machine learning and bioinformatics.

**Yousong Peng** is a professor at Hunan University. His research interests are systems medicine and bioinformatics for infectious diseases.

**Aiping Wu** is a principal investigator at Suzhou Institute of Systems Medicine, Center for Systems Medicine, Chinese Academy of Medical Sciences & Peking Union Medical College, Suzhou, Jiangsu, China. His research interests are deep learning and bioinformatics.

**Taijiao Jiang** is a principal investigator at Guangzhou Laboratory. His research interests are systems medicine and bioinformatics for infectious diseases.
