# Supplementary material for: PREDAC-CNN: predicting antigenic clusters of seasonal influenza A viruses with convolutional neural network
Source: Brief Bioinform. 2024 Feb 10;25(2):bbae033. doi: 10.1093/bib/bbae033 (PMC10859661; doi:10.1093/bib/bbae033)
Supplement: SupplementaryMaterial_final_bbae033 [file supplementarymaterial_final_bbae033.docx]

PREDAC-CNN: predicting antigenic clusters of seasonal influenza A viruses with convolutional neural network

Jing Meng^1#^, Jingze Liu^1#^, Wenkai Song^2#^, Honglei Li^3^, Jiangyuan Wang^4^, Le Zhang^2^, Yousong Peng^5*^, Aiping Wu^1*^, Taijiao Jiang^1, 4, 6*^

1 State Key Laboratory of Common Mechanism Research for Major Diseases，Suzhou Institute of Systems Medicine, Chinese Academy of Medical Sciences & Peking Union Medical College, Suzhou 215123, Jiangsu, China

2 College of Computer Science, Sichuan University, Chengdu 610065, China

3 Beijing Cloudna Technology Company, Limited, Beijing100029, China

4 Guangzhou National Laboratory, Guangzhou 510005, China

5 College of Biology, Hunan University, Changsha 410082, China

6 State Key Laboratory of Respiratory Disease, the First Affiliated Hospital of Guangzhou Medical University, Guangzhou Medical University, Guangzhou, 510120, China

# These authors contributed equally: Jing Meng, Jingze Liu, Wenkai Song.

* Corresponding author

Contact: [jiang_taijiao@gzlab.ac.cn](mailto:taijiaobioinfor@ism.cams.cn%20)  [wap@ism.cams.cn](mailto:wap@ism.cams.cn) pys2013@hnu.edu.cn

**Data Collection**

**HA1 sequence of influenza A/H3N2 and A/H1N1 viruses**

(1) NCBI Influenza Virus Database (http://www.ncbi.nlm.nih.gov/genomes/FLU/Database/)

(2) Global Initiative on Sharing All Influenza Data (<http://platform.gisaid.org/>)

(3) Influenza Research Database

**HI assay data of influenza A/H3N2 and A/H1N1 viruses**

1. Organization: Public Health Surveillance from for New Zealand.

Access: https://surv.esr.cri.nz/PDF_surveillance/Virology/FluVac/

Detail:

Recommendation for the influenza vaccine composition 2014

Recommendation for the influenza vaccine composition 2015

Recommendation for the influenza vaccine composition 2016

Recommendation for the influenza vaccine composition 2018

Recommendation for the influenza vaccine composition 2019

Recommendation for the influenza vaccine composition 2020

Recommendation for the influenza vaccine composition 2021

Recommendation for the influenza vaccine composition 2022

Recommendation for the influenza vaccine composition 2023

1. Organization: European Centre for Disease Prevention and Control.
   Access: http://www.ecdc.europa.eu/en/PUBLICATIONS/surveillance_reports/Pages/index.aspx

Detail:

Influenza virus characterization. Summary Europe, February 2014

Influenza virus characterization. Summary Europe, March 2014

Influenza virus characterization. Summary Europe, April 2014

Influenza virus characterization. Summary Europe, May 2014

Influenza virus characterization. Summary Europe, June 2014

Influenza virus characterization. Summary Europe, July 2014

Influenza virus characterization. Summary Europe, September 2014

Influenza virus characterization. Summary Europe, November 2014

Influenza virus characterization. Summary Europe, December 2014

Influenza virus characterization. Summary Europe, February 2015

Influenza virus characterization. Summary Europe, March 2015

Influenza virus characterization. Summary Europe, April 2015

Influenza virus characterization. Summary Europe, May 2015

Influenza virus characterization. Summary Europe, June 2015

Influenza virus characterization. Summary Europe, July 2015

Influenza virus characterization. Summary Europe, September 2015

Influenza virus characterization. Summary Europe, November 2015

Influenza virus characterization. Summary Europe, December 2015

Influenza virus characterization. Summary Europe, February 2016

Influenza virus characterization. Summary Europe, March 2016

Influenza virus characterization. Summary Europe, May 2016

Influenza virus characterization. Summary Europe, June 2016

Influenza virus characterization. Summary Europe, July 2016

Influenza virus characterization. Summary Europe, September 2016

Influenza virus characterization. Summary Europe, July 2017

Influenza virus characterization. Summary Europe, September 2017

Influenza virus characterization. Summary Europe, November 2017

Influenza virus characterization. Summary Europe, December 2017

Influenza virus characterization. Summary Europe, March 2018

Influenza virus characterization. Summary Europe, May 2018

Influenza virus characterization. Summary Europe, June 2018

Influenza virus characterization. Summary Europe, September 2018

Influenza virus characterization. Summary Europe, October 2018

Influenza virus characterization. Summary Europe, November 2018

Influenza virus characterization. Summary Europe, December 2018

Influenza virus characterization. Summary Europe, March 2019

Influenza virus characterization. Summary Europe, April 2019

Influenza virus characterization. Summary Europe, May 2019

Influenza virus characterization. Summary Europe, June 2019

Influenza virus characterization. Summary Europe, September 2019

Influenza virus characterization. Summary Europe, October 2019

Influenza virus characterization. Summary Europe, November 2019

Influenza virus characterization. Summary Europe, December 2019

Influenza virus characterization. Summary Europe, February 2020

Influenza virus characterization. Summary Europe, March 2020

Influenza virus characterization. Summary Europe, April 2020

Influenza virus characterization. Summary Europe, May 2020

Influenza virus characterization. Summary Europe, June 2020

Influenza virus characterization. Summary Europe, July 2020

Influenza virus characterization. Summary Europe, September 2020

Influenza virus characterization. Summary Europe, October 2020

Influenza virus characterization. Summary Europe, November 2020

Influenza virus characterization. Summary Europe, December 2020

Influenza virus characterization. Summary Europe, February 2021

Influenza virus characterization. Summary Europe, March 2021

Influenza virus characterization. Summary Europe, May 2021

Influenza virus characterization. Summary Europe, April 2021

Influenza virus characterization. Summary Europe, June 2021

1. Organization: U.S. Food and Drug Administration.

Access:

http://www.fda.gov/AdvisoryCommittees/CommitteesMeetingMaterials/BloodVaccinesandOtherBiologics/VaccinesandRelatedBiologicalProductsAdvisoryCommittee/default.htm.

Detail:

Information for the Vaccines and Related Biological Products Advisory Committee. March 9, 2017
Information for the Vaccines and Related Biological Products Advisory Committee. October 4, 2017
Information for the Vaccines and Related Biological Products Advisory Committee. March 1, 2018
Information for the Vaccines and Related Biological Products Advisory Committee. October 3, 2018
Information for the Vaccines and Related Biological Products Advisory Committee. March 5, 2021

Information for the Vaccines and Related Biological Products Advisory Committee. September 30, 2021

Information for the Vaccines and Related Biological Products Advisory Committee. March 3, 2022

Information for the Vaccines and Related Biological Products Advisory Committee. October 6, 2022

Information for the Vaccines and Related Biological Products Advisory Committee. March 7, 2023

1. Organization: World Health Organization.

Access: <http://www.who.int/wer/en/>

Detail:

Weekly Epidemiological Record 2016 No.1 ~ No.51-52

Weekly Epidemiological Record 2017 No.1 ~ No.51-52

Weekly Epidemiological Record 2018 No.1 ~ No.51-52

Weekly Epidemiological Record 2019 No.1 ~ No.51-52

Weekly Epidemiological Record 2020 No.1 ~ No.51-52

Weekly Epidemiological Record 2021 No.1 ~ No.51-52

Recommended composition of influenza virus vaccines for use in the 2015-2016 northern hemisphere influenza season

Recommended composition of influenza virus vaccines for use in the 2016 southern hemisphere influenza season

Recommended composition of influenza virus vaccines for use in the 2016-2017 northern hemisphere influenza season

Recommended composition of influenza virus vaccines for use in the 2017 southern hemisphere influenza season

Recommended composition of influenza virus vaccines for use in the 2017-2018 northern hemisphere influenza season

Recommended composition of influenza virus vaccines for use in the 2018 southern hemisphere influenza season

Recommended composition of influenza virus vaccines for use in the 2018-2019 northern hemisphere influenza season

Recommended composition of influenza virus vaccines for use in the 2019 southern hemisphere influenza season

Recommended composition of influenza virus vaccines for use in the 2019-2020 northern hemisphere influenza season

Recommended composition of influenza virus vaccines for use in the 2020 southern hemisphere influenza season

Recommended composition of influenza virus vaccines for use in the 2020-2021 northern hemisphere influenza season

Recommended composition of influenza virus vaccines for use in the 2021 southern hemisphere influenza season

Recommended composition of influenza virus vaccines for use in the 2021-2022 northern hemisphere influenza season

Recommended composition of influenza virus vaccines for use in the 2022 southern hemisphere influenza season

Recommended composition of influenza virus vaccines for use in the 2022-2023 northern hemisphere influenza season

Recommended composition of influenza virus vaccines for use in the 2023 southern hemisphere influenza season

Recommended composition of influenza virus vaccines for use in the 2023-2024 northern hemisphere influenza season

1. Organization: National Institute for Medical Research

Access: <https://www.crick.ac.uk/partnerships/worldwide-influenza-centre/annual-and-interim-reports>

Detail:

Interim Report February 2023

Interim Report September 2022

Interim Report February 2022

Interim Report September 2021

Interim Report February 2021

Interim Report September 2020

Interim Report February 2020

Interim Report September 2019

Interim Report February 2019

Interim Report September 2018

Interim Report February 2018

Interim Report September 2017

Interim Report February 2017

Interim Report September 2016

Interim Report February 2016

Interim Report September 2015

Interim Report February 2015

Interim Report September 2014

Interim Report February 2014

1. Published papers:

Liu F, Levine M Z. Heterologous Antibody Responses Conferred by A (H3N2) Variant and Seasonal Influenza Vaccination Against Newly Emerged 2016–2018 A (H3N2) Variant Viruses in Healthy Persons [J]. Clinical Infectious Diseases, 2020, 71(12): 3061-3070.

**The multiple entries correspond to each of the six physicochemical properties in the AAindex1 database.**

1. Accessible surface:

H CHOC760101 D Residue accessible surface area in tripeptide (Chothia, 1976)

H CHOC760102 D Residue accessible surface area in folded protein (Chothia, 1976)

H JANJ780101 D Average accessible surface area (Janin et al., 1978)

H RADA880106 D Accessible surface area (Radzicka-Wolfenden, 1988)

H ROSG850101 D Mean area buried on transfer (Rose et al., 1985)

H ROSG850102 D Mean fractional area loss (Rose et al., 1985)

1. Charge:

H CHAM830107 D A parameter of charge transfer capability (Charton-Charton, 1983)

H CHAM830108 D A parameter of charge transfer donor capability (Charton-Charton, 1983)

H FAUJ880111 D Positive charge (Fauchere et al., 1988)

H KLEP840101 D Net charge (Klein et al., 1984)

1. Hydrophobicity:

H ARGP820101 D Hydrophobicity index (Argos et al., 1982)

H CIDH920101 D Normalized hydrophobicity scales for alpha-proteins (Cid et al., 1992)

H EISD840101 D Consensus normalized hydrophobicity scale (Eisenberg, 1984)

H WILM950101 D Hydrophobicity coefficient in RP-HPLC, C18 with 0.1% TFA/MeCN/H2O (Wilce et al. 1995)

H BLAS910101 D Scaled side chain hydrophobicity values (Black-Mould, 1991)

H JURD980101 D Modified Kyte-Doolittle hydrophobicity scale (Juretic et al., 1998)

1. Hyindex (indicating if an amino acid can be hydrogen-bond donor and/or acceptor) :

H FAUJ880109 D Number of hydrogen bond donors (Fauchere et al., 1988)

1. Polarity:

H GRAR740102 D Polarity (Grantham, 1974)

H RADA880108 D Mean polarity (Radzicka-Wolfenden, 1988)

H ZIMJ680103 D Polarity (Zimmerman et al., 1968)

1. Volume:

H BIGC670101 D Residue volume (Bigelow, 1967)

H BULH740102 D Apparent partial specific volume (Bull-Breese, 1974)

H CHOC750101 D Average volume of buried residue (Chothia, 1975)

H COHE430101 D Partial specific volume (Cohn-Edsall, 1943)

H FAUJ880103 D Normalized van der Waals volume (Fauchere et al., 1988)

H GOLD730102 D Residue volume (Goldsack-Chalifoux, 1973)

H GRAR740103 D Volume (Grantham, 1974)

H KRIW790103 D Side chain volume (Krigbaum-Komoriya, 1979)

H TSAJ990101 D Volumes including the crystallographic waters using the ProtOr (Tsai et al., 1999)

H TSAJ990102 D Volumes not including the crystallographic waters using the ProtOr (Tsai et al., 1999)

H HARY940101 D Mean volumes of residues buried in protein interiors (Harpaz et al., 1994)

**Feature Encodings**

To investigate how feature encoding affects the models' performance, we designed three additional feature encodings: AAindex-PCA [1], ESM-2 [2], and iFeatureOmega [3].

1. AAindex-PCA employed principal component analysis (PCA) to address potential high dependencies among the 553 physicochemical properties of amino acids in the AAindex1 database [1]. The first 11 principal components in PCA were utilized to explain approximately 91% of the total variance. The resulting embedding dimension for each amino acid is 11. The input matrix for the paired HA1 sequences (sequence 1 and sequence 2) is constructed based on this embedding, creating a spatially oriented representation of the HA1 sequence. In total, there are 24 rows (12 rows for each sequence) and 329 columns (for influenza A/H3N2 viruses) / 327 columns (for influenza A/H1N1 viruses) in the input matrix. Within each column, the first and last 12 rows represent the amino acid covering the site and its AAindex-PCA embedding for sequence 1 and sequence 2, respectively.
2. The pre-trained ESM-2 model was developed by training a deep learning (transformer protein language) model with up to 15 billion parameters using experimental and high-quality predicted structures [2]. This state-of-the-art general-purpose ESM-2 model can directly predict protein structure, function, and other properties from individual sequences. We inputted the HA1 sequences of influenza A/H3N2 and A/H1N1 viruses into the ESM-2 model. The output from the last layer of the transformer model (esm2_t6_8M_UR50D) was employed as the feature encoding for each amino acid in the HA1 sequence. For each amino acid, the embedding dimension is 320. The input matrix for the paired HA1 sequences (sequence 1 and sequence 2) is constructed based on this embedding, creating a spatially oriented representation of the HA1 sequence. In total, there are 640 rows (320 rows for each sequence) and 329 columns (for influenza A/H3N2 viruses) / 327 columns (for influenza A/H1N1 viruses) in the input matrix. Within each column, the first and last 320 rows represent the ESM-2 embeddings for the amino acid covering the site in sequence 1 and sequence 2, respectively.
3. iFeatureOmega utilizes the AAindex database to quantify the physicochemical properties of individual amino acids [3]. The resulting embedding dimension for each amino acid is 531. The input matrix for the paired HA1 sequences (sequence 1 and sequence 2) is constructed based on this embedding, creating a spatially oriented representation of the HA1 sequence. In total, there are 1064 rows (532 rows for each sequence) and 329 columns (for influenza A/H3N2 viruses) / 327 columns (for influenza A/H1N1 viruses) in the input matrix. Within each column, the first and last 532 rows represent the amino acid covering the site and its iFeatureOmega embeddings for sequence 1 and sequence 2, respectively.

**Supplementary Figures**

**
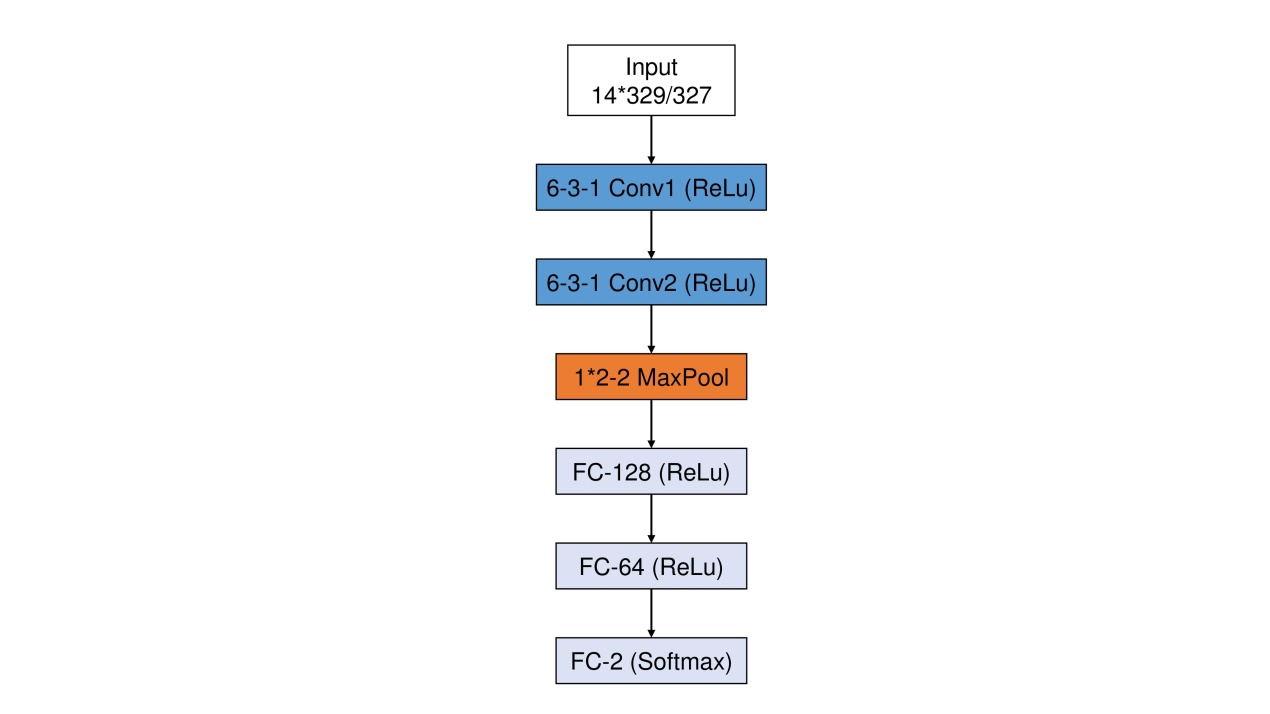
**

**Supplementary Figure S1.** **The architecture of the CNN model.**

The input matrix corresponding to the paired strains is of size 14x329 for influenza A/H3N2 viruses and 14x327 for influenza A/H1N1 viruses, respectively. '6-3-1 Conv1' represents the first one-dimensional convolutional layer with 6 filters of size 3 and a stride of 1. '1x2-2 MaxPool' represents a downsampling layer over 1x2 regions with a stride of 2. 'FC-128,' 'FC-64,' and 'FC-2' denote fully connected linear layers with 128, 64, and 2 neurons, respectively. The number 2 corresponds to the two classes (antigenically distinct and antigenically similar) that represent the antigenic relationship of the paired strains. 'ReLU' and 'Softmax' represent different activation functions.

**
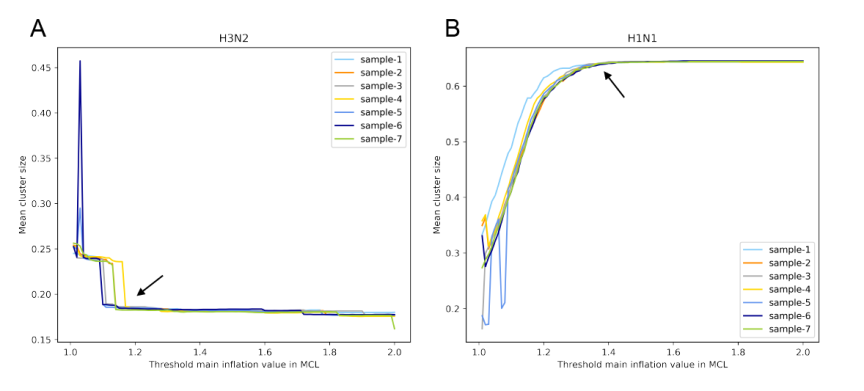
**

**Supplementary Figure S2.** **Cluster size curve for inferring antigenic clusters using the MCL method.**

The mean cluster size represents the probability that two randomly selected viruses belong to the same predicted antigenic cluster [4]. The threshold, denoted by the main inflation value at the start of the first plateau on the cluster size curve (indicated by an arrow), was utilized for inferring antigenic clusters.

**
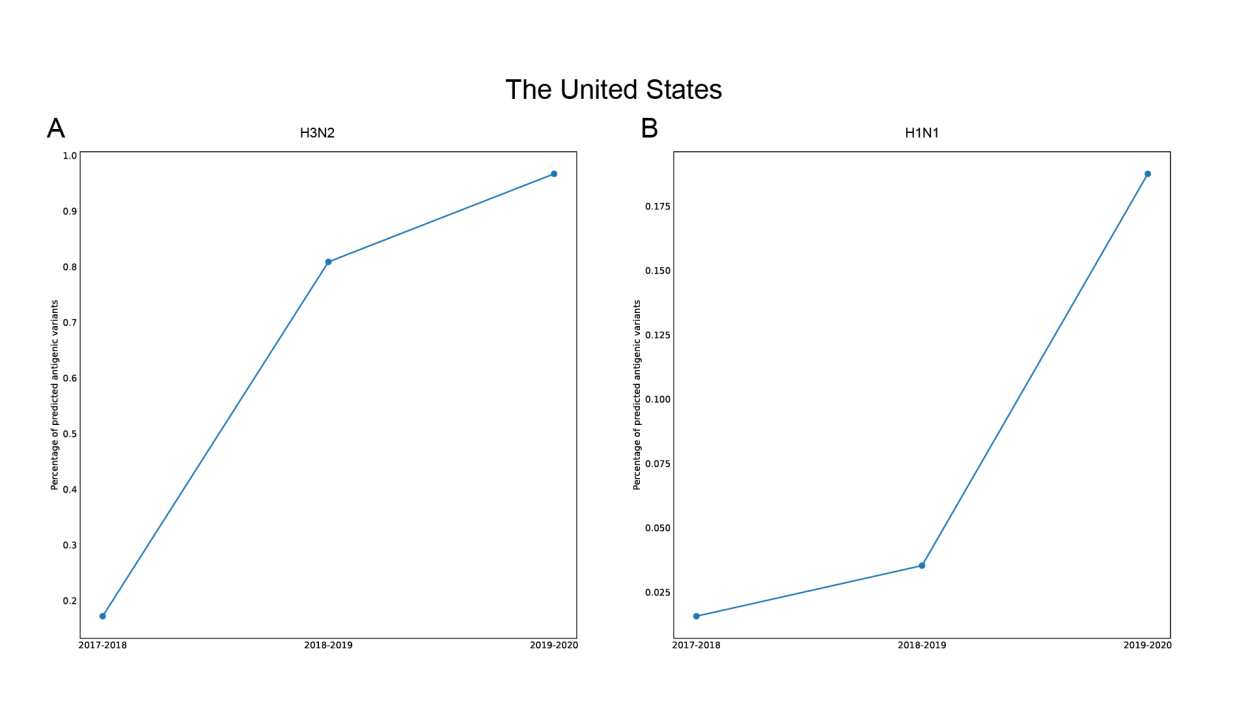
**

**Supplementary Figure S3.** **The percentage of predicted antigenic variants in three influenza seasons in the United States.**

Antigenic variants in a given influenza season were circulating strains that were antigenically distinct from the WHO-recommended vaccine strain. (A) The percentage of predicted antigenic variants for influenza A/H3N2 viruses. (B) The percentage of predicted antigenic variants for influenza A/H1N1 viruses.

**
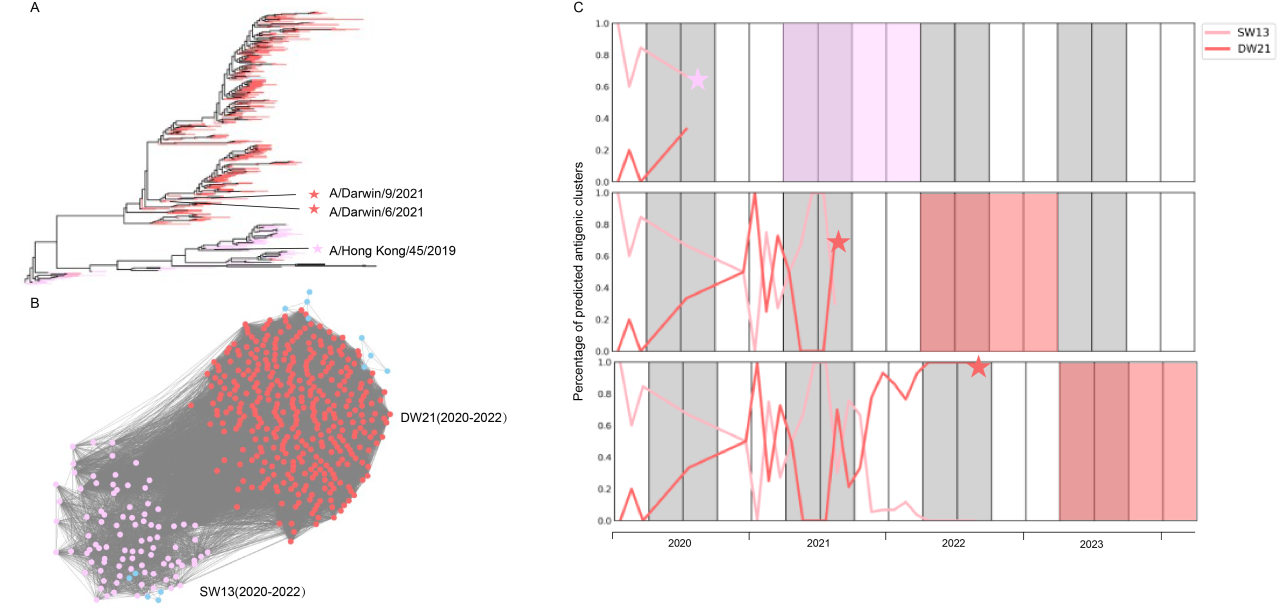
**

**Supplementary Figure S4.** **Vaccine strain recommendations against influenza A/H1N1 viruses for three influenza seasons in the Southern Hemisphere.**

(A) Genetic evolution of circulating strains, with WHO-recommended vaccine strains represented by five-pointed stars. (B) Antigenic evolution of circulating strains, with antigenic clusters named after abbreviations of the WHO-recommended earliest vaccine strains contained in the clusters. (C) Vaccine strain recommendations season by season based on the percentage of predicted antigenic clusters. Five-pointed stars indicate the dates (late September) for vaccine strain recommendations. Gray backgrounds indicate winter seasons in the Southern Hemisphere. Colored backgrounds indicate seasons when predicted vaccine strains were to be used. Different colored lines correspond to different antigenic clusters.

**Supplementary Tables**

**Supplementary Table S1.** Feature dictionary for influenza A/H3N2 viruses: six physicochemical features with corresponding optimal entries and continuous encodings for 20 amino acids.

|  | Accessible surface | Charge | Hydrophobicity | Hyindex | Polarity | Volume | Continuous encoding |
| --- | --- | --- | --- | --- | --- | --- | --- |
| A | 0.088608 | 0 | 0.0125 | 0 | 0 | 0.169461 | 0 |
| R | 0.911392 | 1 | -0.17708 | 0.25 | 1 | 0.725749 | 0.05 |
| N | 0.56962 | 0 | 0.052083 | 1 | 0.065 | 0.38982 | 0.1 |
| D | 0.405063 | -1 | -0.04167 | 1 | 0.955769 | 0.304192 | 0.15 |
| C | 0.012658 | 0 | 0.102083 | 0 | 0.028462 | 0.313772 | 0.2 |
| Q | 0.670886 | 0 | 0.064583 | 1 | 0.067885 | 0.531138 | 0.25 |
| E | 0.392405 | -1 | -0.02083 | 0.5 | 0.959615 | 0.482036 | 0.3 |
| G | 0.063291 | 0 | 0.04375 | 0 | 0 | 0 | 0.35 |
| H | 0.316456 | 1 | -0.46667 | 0.5 | 0.992308 | 0.554491 | 0.4 |
| I | 0 | 0 | 0.725 | 0 | 0.0025 | 0.649701 | 0.45 |
| L | 0.063291 | 0 | 0.729167 | 0 | 0.0025 | 0.649701 | 0.5 |
| K | 1 | 1 | -0.3375 | 0.25 | 0.951923 | 0.692216 | 0.55 |
| M | 0.164557 | 0 | 0.04375 | 0 | 0.0275 | 0.611976 | 0.6 |
| F | 0.075949 | 0 | 1 | 0 | 0.006731 | 0.772455 | 0.65 |
| P | 0.405063 | 0 | 0.147917 | 0 | 0.030385 | 0.372455 | 0.7 |
| S | 0.329114 | 0 | -0.12917 | 1 | 0.032115 | 0.171856 | 0.75 |
| T | 0.367089 | 0 | 0.135417 | 1 | 0.031923 | 0.348503 | 0.8 |
| W | 0.177215 | 0 | 0.477083 | 0.25 | 0.040385 | 1 | 0.85 |
| Y | 0.531646 | 0 | 0.39375 | 1 | 0.030962 | 0.796407 | 0.95 |
| V | 0 | 0 | 0.33125 | 0 | 0.0025 | 0.487425 | 1 |

**Supplementary Table S2.** Feature dictionary for influenza A/H1N1 viruses: six physicochemical features with corresponding optimal entries and continuous encodings for 20 amino acids.

|  | Accessible surface | Charge | Hydrophobicity | Hyindex | Polarity | Volume | Continuous encoding |
| --- | --- | --- | --- | --- | --- | --- | --- |
| A | 0.140571 | 0 | 0.0125 | 0 | 0.395062 | 0.189003 | 0 |
| R | 0.905143 | 1 | -0.17708 | 0.25 | 0.691358 | 0.721649 | 0.05 |
| N | 0.509714 | 0 | 0.052083 | 1 | 0.82716 | 0.403436 | 0.1 |
| D | 0.515429 | -1 | -0.04167 | 1 | 1 | 0.274914 | 0.15 |
| C | 0 | 0 | 0.102083 | 0 | 0.074074 | 0.306529 | 0.2 |
| Q | 0.608 | 0 | 0.064583 | 1 | 0.691358 | 0.554639 | 0.25 |
| E | 0.602286 | -1 | -0.02083 | 0.5 | 0.91358 | 0.426116 | 0.3 |
| G | 0.102857 | 0 | 0.04375 | 0 | 0.506173 | 0 | 0.35 |
| H | 0.402286 | 1 | -0.46667 | 0.5 | 0.679012 | 0.542955 | 0.4 |
| I | 0.083429 | 0 | 0.725 | 0 | 0.037037 | 0.642611 | 0.45 |
| L | 0.138286 | 0 | 0.729167 | 0 | 0 | 0.642611 | 0.5 |
| K | 1 | 1 | -0.3375 | 0.25 | 0.790123 | 0.687285 | 0.55 |
| M | 0.205714 | 0 | 0.04375 | 0 | 0.098765 | 0.646735 | 0.6 |
| F | 0.114286 | 0 | 1 | 0 | 0.037037 | 0.793814 | 0.65 |
| P | 0.411429 | 0 | 0.147917 | 0 | 0.382716 | 0.287972 | 0.7 |
| S | 0.302857 | 0 | -0.12917 | 1 | 0.530864 | 0.201374 | 0.75 |
| T | 0.337143 | 0 | 0.135417 | 1 | 0.45679 | 0.352577 | 0.8 |
| W | 0.219429 | 0 | 0.477083 | 0.25 | 0.061728 | 1 | 0.85 |
| Y | 0.453714 | 0 | 0.39375 | 1 | 0.160494 | 0.806185 | 0.95 |
| V | 0.093714 | 0 | 0.33125 | 0 | 0.123457 | 0.491408 | 1 |

**Supplementary Table S3.** Performance comparison of PREDAC-CNN and its five competitors on subset_1 of influenza A/H3N2 viruses.

| Model | Training time  (s) | Testing time  (s) | Memory usage  (GB) | Accuracy | Recall | Precision | F_1_ score |
| --- | --- | --- | --- | --- | --- | --- | --- |
| PREDAC-CNN | 100 | 34 | 6.249 | 0.966 | 0.970 | 0.970 | **0.970** |
| IAV-CNN | 574 | 46 | 9.831 | 0.689 | 0.485 | 0.610 | 0.540 |
| PREDAC-H3 | 41 | 22 | 0.506 | 0.759 | 0.911 | 0.733 | 0.813 |
| PREDAV-FluA | 43.964 | 0.036 | 1.045 | 0.778 | 0.761 | 0.836 | 0.797 |
| Lee | 0 | 14 | 0.272 | 0.573 | 0.999 | 0.573 | 0.728 |
| Lees | 54.993 | 0.007 | 0.273 | 0.795 | 0.815 | 0.825 | 0.820 |

**Supplementary Table S4.** Performance comparison of PREDAC-CNN and its five competitors on subset_2 of influenza A/H3N2 viruses.

| Model | Training time  (s) | Testing time  (s) | Memory usage  (GB) | Accuracy | Recall | Precision | F_1_ score |
| --- | --- | --- | --- | --- | --- | --- | --- |
| PREDAC-CNN | 110 | 35 | 7.492 | 0.972 | 0.977 | 0.971 | **0.974** |
| IAV-CNN | 978 | 49 | 9.829 | 0.674 | 0.426 | 0.617 | 0.504 |
| PREDAC-H3 | 41 | 23 | 0.559 | 0.758 | 0.914 | 0.715 | 0.803 |
| PREDAV-FluA | 42.970 | 0.030 | 1.045 | 0.782 | 0.789 | 0.803 | 0.796 |
| Lee | 0 | 14 | 0.272 | 0.539 | 1.000 | 0.539 | 0.700 |
| Lees | 52.993 | 0.007 | 0.273 | 0.816 | 0.856 | 0.812 | 0.834 |

**Supplementary Table S5.** Performance comparison of PREDAC-CNN and its five competitors on subset_3 of influenza A/H3N2 viruses.

| Model | Training time  (s) | Testing time  (s) | Memory usage  (GB) | Accuracy | Recall | Precision | F_1_ score |
| --- | --- | --- | --- | --- | --- | --- | --- |
| PREDAC-CNN | 108 | 29 | 7.758 | 0.975 | 0.966 | 0.990 | **0.977** |
| IAV-CNN | 868 | 43 | 9.833 | 0.720 | 0.493 | 0.750 | 0.595 |
| PREDAC-H3 | 40 | 21 | 0.543 | 0.764 | 0.921 | 0.728 | 0.813 |
| PREDAV-FluA | 39.967 | 0.033 | 1.045 | 0.809 | 0.792 | 0.855 | 0.822 |
| Lee | 0 | 13 | 0.272 | 0.558 | 1.000 | 0.558 | 0.716 |
| Lees | 50.994 | 0.006 | 0.273 | 0.808 | 0.815 | 0.837 | 0.825 |

**Supplementary Table S6.** Performance comparison of PREDAC-CNN and its five competitors on subset_4 of influenza A/H3N2 viruses.

| Model | Training time  (s) | Testing time  (s) | Memory usage  (GB) | Accuracy | Recall | Precision | F_1_ score |
| --- | --- | --- | --- | --- | --- | --- | --- |
| PREDAC-CNN | 104 | 35 | 8.265 | 0.967 | 0.967 | 0.975 | **0.971** |
| IAV-CNN | 1073 | 43 | 9.827 | 0.663 | 0.486 | 0.632 | 0.550 |
| PREDAC-H3 | 41 | 22 | 0.569 | 0.782 | 0.921 | 0.751 | 0.827 |
| PREDAV-FluA | 41.963 | 0.037 | 1.045 | 0.800 | 0.797 | 0.842 | 0.819 |
| Lee | 0 | 12 | 0.272 | 0.567 | 1.000 | 0.567 | 0.724 |
| Lees | 51.994 | 0.006 | 0.273 | 0.820 | 0.854 | 0.834 | 0.844 |

**Supplementary Table S7.** Performance comparison of PREDAC-CNN and its five competitors on subset_5 of influenza A/H3N2 viruses.

| Model | Training time  (s) | Testing time  (s) | Memory usage  (GB) | Accuracy | Recall | Precision | F_1_ score |
| --- | --- | --- | --- | --- | --- | --- | --- |
| PREDAC-CNN | 124 | 30 | 7.991 | 0.971 | 0.971 | 0.975 | **0.973** |
| IAV-CNN | 1151 | 40 | 9.839 | 0.665 | 0.481 | 0.696 | 0.569 |
| PREDAC-H3 | 41 | 22 | 0.512 | 0.750 | 0.902 | 0.715 | 0.797 |
| PREDAV-FluA | 39.967 | 0.033 | 1.045 | 0.783 | 0.776 | 0.817 | 0.796 |
| Lee | 0 | 12 | 0.272 | 0.545 | 1.000 | 0.545 | 0.706 |
| Lees | 51.990 | 0.010 | 0.273 | 0.798 | 0.816 | 0.814 | 0.815 |

**Supplementary Table S8.** Performance comparison of PREDAC-CNN and its five competitors on subset_1 of influenza A/H1N1 viruses.

| Model | Training time  (s) | Testing time  (s) | Memory usage  (GB) | Accuracy | Recall | Precision | F_1_ score |
| --- | --- | --- | --- | --- | --- | --- | --- |
| PREDAC-CNN | 73 | 17 | 4.249 | 0.886 | 0.864 | 0.838 | **0.851** |
| IAV-CNN | 30 | 17 | 5.183 | 0.689 | 0.485 | 0.610 | 0.540 |
| PREDAC-H1 | 8 | 7 | 0.151 | 0.669 | 0.561 | 0.561 | 0.561 |
| PREDAV-FluA | 10.956 | 0.044 | 1.042 | 0.657 | 0.485 | 0.552 | 0.516 |
| Lee | 0 | 12 | 0.269 | 0.377 | 1.000 | 0.377 | 0.548 |
| Lees | 19.994 | 0.006 | 0.269 | 0.634 | 0.515 | 0.515 | 0.515 |

**Supplementary Table S9.** Performance comparison of PREDAC-CNN and its five competitors on subset_2 of influenza A/H1N1 viruses.

| Model | Training time  (s) | Testing time  (s) | Memory usage  (GB) | Accuracy | Recall | Precision | F_1_ score |
| --- | --- | --- | --- | --- | --- | --- | --- |
| PREDAC-CNN | 55 | 16 | 4.275 | 0.880 | 0.882 | 0.822 | **0.851** |
| IAV-CNN | 36 | 12 | 5.177 | 0.674 | 0.426 | 0.617 | 0.504 |
| PREDAC-H1 | 9 | 7 | 0.158 | 0.646 | 0.529 | 0.545 | 0.537 |
| PREDAV-FluA | 11.955 | 0.045 | 1.042 | 0.720 | 0.588 | 0.656 | 0.620 |
| Lee | 0 | 15 | 0.269 | 0.394 | 1.000 | 0.391 | 0.562 |
| Lees | 25.995 | 0.005 | 0.269 | 0.623 | 0.456 | 0.517 | 0.484 |

**Supplementary Table S10.** Performance comparison of PREDAC-CNN and its five competitors on subset_3 of influenza A/H1N1 viruses.

| Model | Training time  (s) | Testing time  (s) | Memory usage  (GB) | Accuracy | Recall | Precision | F_1_ score |
| --- | --- | --- | --- | --- | --- | --- | --- |
| PREDAC-CNN | 39 | 16 | 4.021 | 0.869 | 0.863 | 0.829 | **0.846** |
| IAV-CNN | 48 | 13 | 5.189 | 0.720 | 0.493 | 0.750 | 0.595 |
| PREDAC-H1 | 7 | 7 | 0.164 | 0.686 | 0.603 | 0.629 | 0.615 |
| PREDAV-FluA | 11.971 | 0.029 | 1.042 | 0.697 | 0.534 | 0.672 | 0.595 |
| Lee | 0 | 13 | 0.269 | 0.417 | 1.000 | 0.417 | 0.589 |
| Lees | 23.995 | 0.005 | 0.269 | 0.680 | 0.589 | 0.623 | 0.606 |

**Supplementary Table S11.** Performance comparison of PREDAC-CNN and its five competitors on subset_4 of influenza A/H1N1 viruses.

| Model | Training time  (s) | Testing time  (s) | Memory usage  (GB) | Accuracy | Recall | Precision | F_1_ score |
| --- | --- | --- | --- | --- | --- | --- | --- |
| PREDAC-CNN | 34 | 20 | 4.163 | 0.840 | 0.838 | 0.795 | **0.816** |
| IAV-CNN | 36 | 13 | 5.183 | 0.663 | 0.486 | 0.632 | 0.550 |
| PREDAC-H1 | 8 | 6 | 0.155 | 0.663 | 0.662 | 0.590 | 0.624 |
| PREDAV-FluA | 11.965 | 0.035 | 1.042 | 0.674 | 0.568 | 0.627 | 0.596 |
| Lee | 0 | 13 | 0.269 | 0.429 | 1.000 | 0.425 | 0.597 |
| Lees | 24.996 | 0.004 | 0.269 | 0.697 | 0.527 | 0.684 | 0.595 |

**Supplementary Table S12.** Performance comparison of PREDAC-CNN and its five competitors on subset_5 of influenza A/H1N1 viruses.

| Model | Training time  (s) | Testing time  (s) | Memory usage  (GB) | Accuracy | Recall | Precision | F_1_ score |
| --- | --- | --- | --- | --- | --- | --- | --- |
| PREDAC-CNN | 33 | 16 | 2.220 | 0.875 | 0.877 | 0.855 | **0.866** |
| IAV-CNN | 63 | 13 | 5.180 | 0.665 | 0.481 | 0.696 | 0.569 |
| PREDAC-H1 | 9 | 7 | 0.150 | 0.648 | 0.580 | 0.627 | 0.603 |
| PREDAV-FluA | 11.963 | 0.037 | 1.042 | 0.659 | 0.494 | 0.678 | 0.571 |
| Lee | 0 | 10 | 0.269 | 0.466 | 1.000 | 0.463 | 0.633 |
| Lees | 19.995 | 0.005 | 0.269 | 0.659 | 0.432 | 0.714 | 0.538 |

**Supplementary Table S13.** AUPRC (Area Under Precision-Recall Receiver Operating Characteristic) values for PREDAC-CNN and its five competitors on 17 testing subsets of influenza A/H3N2 viruses.

| Year | AUPRC | | | | | |
| --- | --- | --- | --- | --- | --- | --- |
|  | PREDAC-CNN | IAV-CNN | PREDAC-H3 | PREDAV-FluA | Lee | Lees |
| 2006 | **0.463** | 0.248 | 0.193 | 0.305 | 0.268 | 0.236 |
| 2007 | **0.934** | 0.630 | 0.157 | 0.755 | 0.427 | 0.610 |
| 2008 | 0.514 | 0.605 | 0.252 | 0.452 | **0.634** | 0.625 |
| 2009 | 0.981 | 0.980 | 0.981 | **0.997** | 0.977 | 0.995 |
| 2010 | **0.951** | 0.822 | 0.710 | 0.848 | 0.805 | 0.851 |
| 2011 | **0.781** | 0.174 | 0.227 | 0.644 | 0.184 | 0.462 |
| 2012 | NA | NA | NA | NA | NA | NA |
| 2013 | **0.638** | 0.427 | 0.420 | 0.367 | 0.442 | 0.512 |
| 2014 | **0.842** | 0.626 | 0.543 | 0.751 | 0.667 | 0.566 |
| 2015 | 0.975 | **1.000** | 0.773 | 0.900 | **1.000** | **1.000** |
| 2016 | **0.754** | 0.621 | 0.479 | 0.683 | 0.579 | 0.621 |
| 2017 | **0.911** | 0.832 | 0.677 | 0.761 | 0.850 | 0.888 |
| 2018 | **1.000** | 0.962 | 0.824 | 0.966 | 0.969 | 0.991 |
| 2019 | 0.925 | **0.955** | 0.807 | 0.939 | 0.946 | 0.928 |
| 2020 | **0.719** | 0.554 | 0.450 | 0.516 | 0.572 | 0.539 |
| 2021 | 0.794 | 0.992 | 0.971 | **0.997** | 0.995 | 0.994 |
| 2022 | 0.846 | 0.936 | 0.767 | **0.948** | 0.925 | 0.932 |

**Supplementary Table S14.** AUPRC (Area Under Precision-Recall Receiver Operating Characteristic) values for PREDAC-CNN and its five competitors on 17 testing subsets of influenza A/H1N1 viruses.

| Year | AUPRC | | | | | |
| --- | --- | --- | --- | --- | --- | --- |
|  | PREDAC-CNN | IAV-CNN | PREDAC-H1 | PREDAV-FluA | Lee | Lees |
| 2006 | 0.555 | **0.824** | 0.769 | 0.596 | 0.763 | 0.716 |
| 2007 | **0.916** | 0.835 | 0.665 | 0.588 | 0.741 | 0.535 |
| 2008 | **0.907** | 0.773 | 0.604 | 0.777 | 0.482 | 0.682 |
| 2009 | 0.266 | 0.503 | 0.595 | 0.545 | 0.509 | **0.660** |
| 2010 | **0.417** | 0.052 | 0.121 | 0.059 | 0.080 | 0.105 |
| 2011 | NA | NA | NA | NA | NA | NA |
| 2012 | **0.851** | 0.478 | 0.531 | 0.828 | 0.489 | 0.645 |
| 2013 | 0.487 | 0.585 | 0.250 | 0.581 | **0.606** | 0.529 |
| 2014 | **1.000** | 0.317 | 0.367 | 0.583 | 0.367 | 0.750 |
| 2015 | 0.227 | 0.174 | 0.178 | 0.236 | 0.171 | **0.316** |
| 2016 | **0.917** | 0.345 | 0.322 | 0.478 | 0.396 | 0.778 |
| 2017 | NA | NA | NA | NA | NA | NA |
| 2018 | NA | NA | NA | NA | NA | NA |
| 2019 | **0.723** | 0.670 | 0.559 | 0.518 | 0.582 | 0.465 |
| 2020 | **1.000** | 0.690 | 0.911 | 0.976 | 0.958 | 0.976 |
| 2021 | **0.983** | 0.954 | 0.874 | 0.959 | 0.954 | 0.815 |
| 2022 | **1.000** | **1.000** | **1.000** | **1.000** | **1.000** | **1.000** |

**Supplementary Table S15.** Summary of antigenic clusters of influenza A/H3N2 viruses corresponding to seven samples.

| Cluster | sample-1 | |  | sample-2 | | sample-3 | | sample-4 | | sample-5 | | sample-6 | | sample-7 | |
| --- | --- | --- | --- | --- | --- | --- | --- | --- | --- | --- | --- | --- | --- | --- | --- |
|  | Circulation time | Strains' number |  | Circulation time | Strains' number | Circulation time | Strains' number | Circulation time | Strains' number | Circulation time | Strains' number | Circulation time | Strains' number | Circulation time | Strains' number |
| HK68 | 1968-1972 | 18 |  | 1968-1972 | 19 | 1968-1972 | 17 | 1968-1972 | 19 | 1968-1972 | 18 | 1968-1972 | 19 | 1968-1972 | 19 |
| EN72 | 1972-1975 | 16 |  | 1972-1975 | 15 | 1972-1975 | 16 | 1972-1975 | 14 | 1972-1975 | 15 | 1972-1975 | 15 | 1972-1975 | 14 |
| VI75 | 1975-1976 | 5 |  | 1975-1976 | 5 | 1975-1976 | 7 | 1975-1976 | 5 | 1975-1976 | 5 | 1975-1975 | 4 | 1975-1976 | 5 |
| TX77 | 1976-1978 | 7 |  | 1976-1978 | 7 | 1976-1978 | 6 | 1976-1978 | 7 | 1976-1978 | 7 | 1976-1978 | 6 | 1976-1978 | 7 |
| BK79 | 1978-1988 | 71 |  | 1978-1988 | 69 | 1978-1988 | 68 | 1978-1988 | 67 | 1978-1988 | 72 | 1979-1988 | 49 | 1978-1988 | 67 |
| SI87 | 1987-1991 | 44 |  | 1987-1991 | 43 | 1987-1991 | 43 | 1987-1991 | 42 | 1987-1991 | 42 | 1987-1991 | 43 | 1987-1991 | 43 |
| BE89 | 1989-1993 | 30 |  | 1989-1993 | 30 | 1988-1993 | 31 | 1989-1993 | 30 | 1989-1993 | 30 | 1988-1993 | 31 | 1989-1993 | 30 |
| BE92 | 1991-1997 | 89 |  | 1991-1997 | 88 | 1991-1997 | 84 | 1990-1997 | 92 | 1991-1997 | 90 | 1991-1997 | 90 | 1991-1997 | 89 |
| WU95 | 1993-1999 | 97 |  | 1993-1999 | 98 | 1993-1999 | 101 | 1993-1999 | 96 | 1993-1999 | 96 | 1993-1999 | 97 | 1993-1999 | 97 |
| SY97 | 1996-2005 | 197 |  | 1996-2005 | 205 | 1996-2005 | 206 | 1996-2005 | 219 | 1996-2005 | 209 | 1996-2005 | 185 | 1996-2005 | 204 |
| FU02 | 2002-2010 | 386 |  | 2001-2010 | 382 | 2001-2010 | 381 | 2002-2010 | 382 | 2001-2010 | 378 | 2001-2010 | 382 | 2001-2010 | 381 |
| PE09 | 2009-2016 | 409 |  | 2009-2016 | 395 | 2009-2016 | 396 | 2009-2016 | 398 | 2009-2016 | 393 | 2009-2016 | 400 | 2009-2016 | 395 |
| SW13 | 2012-2022 | 863 |  | 2012-2022 | 874 | 2012-2022 | 873 | 2012-2022 | 870 | 2012-2022 | 879 | 2012-2022 | 871 | 2012-2022 | 871 |
| DW21 | 2020-2023 | 190 |  | 2020-2023 | 188 | 2020-2023 | 187 | 2020-2023 | 189 | 2020-2023 | 188 | 2020-2023 | 190 | 2020-2023 | 194 |

**Supplementary Table S16.** Summary of antigenic clusters of influenza A/H1N1 viruses corresponding to seven samples.

| Cluster | sample-1 | | sample-2 | | sample-3 | | sample-4 | | sample-5 | | sample-6 | | sample-7 | |
| --- | --- | --- | --- | --- | --- | --- | --- | --- | --- | --- | --- | --- | --- | --- |
|  | Circulation time | Strains' number | Circulation time | Strains' number | Circulation time | Strains' number | Circulation time | Strains' number | Circulation time | Strains' number | Circulation time | Strains' number | Circulation time | Strains' number |
| US77 | 1977-1985 | 19 | 1977-1984 | 18 | 1977-1984 | 20 | 1977-1984 | 18 | 1977-1984 | 18 | 1977-1984 | 18 | 1977-1984 | 18 |
| SI86 | 1986-1996 | 23 | 1986-1996 | 23 | 1986-1996 | 23 | 1986-1996 | 23 | 1986-1996 | 23 | 1986-1996 | 23 | 1986-1996 | 23 |
| BE95 | 1995-2008 | 90 | 1995-2008 | 89 | 1995-2008 | 87 | 1995-2008 | 87 | 1995-2008 | 89 | 1995-2009 | 89 | 1995-2009 | 93 |
| SI06 | 2006-2010 | 171 | 2006-2010 | 170 | 2006-2010 | 171 | 2006-2010 | 172 | 2006-2010 | 170 | 2006-2010 | 170 | 2006-2010 | 167 |
| CA09 | 2009-2022 | 2033 | 2009-2022 | 2028 | 2009-2022 | 2030 | 2009-2022 | 2030 | 2009-2022 | 2026 | 2009-2022 | 2024 | 2009-2022 | 2031 |
| VI19 | 2018-2023 | 170 | 2018-2023 | 171 | 2018-2023 | 170 | 2018-2023 | 173 | 2018-2023 | 170 | 2018-2023 | 170 | 2018-2023 | 171 |

**References**

1. Forghani M, Khachay M. Convolutional neural network based approach to in silico non-anticipating prediction of antigenic distance for influenza virus, Viruses 2020;12:1019.

2. Lin Z, Akin H, Rao R et al. Evolutionary-scale prediction of atomic-level protein structure with a language model, science 2023;379:1123-1130.

3. Chen Z, Liu X, Zhao P et al. iFeatureOmega: an integrative platform for engineering, visualization and analysis of features from molecular sequences, structural and ligand data sets, Nucleic acids research 2022;50:W434-W447.

4. Plotkin JB, Chave J, Ashton PS. Cluster analysis of spatial patterns in Malaysian tree species, The American Naturalist 2002;160:629-644.
